# Efficient Cr(VI) Removal from Acidic Wastewater by Tannic-Acid/Fe_3_O_4_-Modified Corn Straw Biochar: Performance and Mechanism

**DOI:** 10.3390/molecules31122169

**Published:** 2026-06-20

**Authors:** Xiaohua Shu, Jiayi Xiao, Huimei Shan, Yunquan Liu, Sanxi Peng

**Affiliations:** 1 Guangxi Key Laboratory of Environmental Pollution Control Theory and Technology, Guilin University of Technology, Guilin 541006, China; sxh9911@glut.edu.cn (X.S.); one9230@126.com (J.X.); 2 Engineering Research Center of Watershed Protection and Green Development, Guilin University of Technology, Guilin 541006, China; 3 Guangxi Engineering Research Center of Comprehensive Treatment for Agricultural Non-Point Source Pollution, Guilin University of Technology, Guilin 541006, China; 4 Modern Industry College of Ecology and Environmental Protection, Guilin University of Technology, Guilin 541006, China; buddychampion@163.com; 5 Collaborative Innovation Center for Water Pollution Control and Water Safety in Karst Area, Guilin University of Technology, Guilin 541006, China; pengsanxi1984@163.com

**Keywords:** acidic wastewater, iron-modified biochar, tannic acid, Cr(VI) removal

## Abstract

The problem of chromium contamination, especially Cr(VI), in acidic wastewater has drawn significant attention, requiring effective and sustainable remediation measures. In this study, tannic-acid/Fe_3_O_4_-modified corn straw biochar (Fe-TA-CSB) is prepared by a grinding-calcination method to remove Cr(VI). The factors influencing the removal effect of Fe-TA-CSB are investigated through static adsorption experiments. The removal mechanism is explored by combining adsorption kinetics, isothermal adsorption, and thermodynamics, as well as characterization methods. The results show that the removal efficiency of Cr(VI) increases with the increase in pH, contact time (*t*), and solid–liquid ratio (*m*/*v*), but decreases with the increase in initial concentration (C_0_). Under optimal conditions of TA/Fe_3_O_4_ mass ratio = 12.5%, pH = 3.0, *m*/*v* = 1.0 g/L, and C_0_ = 10 mg/L, the removal efficiency value is 94.02%, which is approximately 81.44% after four adsorption–desorption cycles. The adsorption behavior is fitted well by the Sips isotherm model and Elovich kinetics model, suggesting the adsorption process of heterogeneous monolayer chemisorption. The removal mechanism of Cr(VI) by Fe-TA-CSB involves electrostatic interaction with Cr(VI), reduction in Cr(VI) to Cr(III) through C–O and Fe(II), and complexation of reduced Cr(III) with the introduced Fe–O and phenolic hydroxyl groups. Fe-TA-CSB is an environmentally friendly and renewable adsorbent with good potential for the treatment of acidic wastewater.

## 1. Introduction

Strongly acidic wastewater containing chromium, produced from chromium ore mining as well as leather tanning and electroplating processing industries, has caused serious pollution to the environment [1,2]. Trivalent chromium (Cr(III)) and hexavalent chromium (Cr(VI)) are the common and stable states of chromium, which have a great difference in acidic wastewater [3]. Compared to Cr(III), Cr(VI) is more toxic, mobile, and soluble, which is the predominant species in acidic wastewater [4]. It has been reported that the concentration of acidic Cr(VI)-polluted wastewater from the electroplating rinsing process exceeds 50 mg/L with low pH values ranging from 0.5 to 4 [5]. This kind of wastewater can directly damage the survival of aquatic organisms, pose significant risks to human health and cause diseases such as skin ulceration, DNA damage, and lung cancer [6,7]. Hence, it is of great urgency and necessity to remove Cr(VI) from acidic industrial wastewater.

According to reported literature, various methods have been used in the treatment of acidic Cr(VI) wastewater, such as adsorption, membrane separation, ion exchange and chemical precipitation [8,9,10]. Among them, adsorption has attracted widespread attention because of its advantages of low cost and simple operation [11]. However, most adsorbents suffer from poor adsorption efficiency and deactivation under acidic conditions due to the strong oxidizing property of Cr(VI) of the practical Cr(VI)-containing wastewater [12]. Therefore, it is important to find appropriate adsorbents with high efficiency and strong stability to remove Cr(VI) from acidic wastewater.

In recent years, biochar has been successfully applied in the removal of Cr(VI) under acidic solution [13]. This is because the positive surface of biochar at low pH enhances electrostatic attraction to anionic Cr(VI), and abundant oxygen-containing functional groups of biochar facilitate the reduction in Cr(VI) at low pH [14]. It is noted that these physicochemical properties of biochar affecting Cr(VI) removal performance are highly dependent on the choice of raw materials [15]. Corn straw, as an abundant agricultural waste resource rich in cellulose, hemicellulose and lignin, is an eco-friendly and cost-effective feedstock for biochar [16]. Past studies have demonstrated that it possesses a relatively high specific surface area and abundant oxygen-containing functional groups such as C–O and C=O groups, making it a promising candidate for Cr(VI) remediation [17]. However, pristine corn straw biochar also exhibits notable limitations, such as insufficient active sites, poor reducibility, and difficulty in recovery from solutions [18]. In order to solve these problems, some researchers have tried to modify biochar by loading iron-based materials with reducibility and magnetism [19]. Among them, Fe_3_O_4_ has become the main iron-based material due to its high chemical stability, excellent chelation behavior, and strong affinity for anions [20]. The Fe_3_O_4_ provides additional properties for biochar, such as increasing surface functional groups, providing more active sites, and granting magnetic separation capability, which promotes the adsorption of Cr(VI). In addition, Fe_3_O_4_ could continuously convert Cr(VI) into Cr(III) through Fe(II)/Fe(III) cycle-mediated electron transfer [21]. Previous studies have demonstrated that Fe_3_O_4_/biochar exhibits good Cr(VI) adsorption ability. Cai et al. [22] found that loading Fe_3_O_4_ increased the removal rate of biochar for Cr(VI) from 44.25% to 96.47%. However, Fe_3_O_4_/biochar materials suffer from problems such as iron particle agglomeration and pore blockage, which hinder the removal process of Cr(VI) [23].

Currently, the modification of Fe_3_O_4_/biochar by introducing active functional groups is regarded as one of the effective methods to enhance its performance [24]. Among numerous functional groups, phenolic hydroxyl groups serve to provide efficient adsorption binding sites for Cr(VI) and show a strong reducing ability for Cr(VI), which are ideal functional groups for enhancing the removal performance of Fe_3_O_4_/biochar for Cr(VI) [25]. So far, various phenolic hydroxyl precursors have been used to prepare phenolic hydroxyl-modified materials. Among them, polyphenols, as natural compounds rich in phenolic hydroxyl groups, have attracted much attention due to their wide sources, diverse structural forms, and green and non-toxic properties [25]. On the one hand, polyphenols can bind to carbon materials through hydrogen bonding. On the other hand, polyphenols can form strong complexes with iron ions through chelation, reducing more Fe^2+^ to promote the conversion of Cr(VI) to Cr(III) [26]. However, different types of polyphenols often have different numbers and chemical structures of phenolic hydroxyl groups, which lead to differences in their binding capabilities to the materials, ultimately affecting the removal of Cr(VI) [27]. Compared with other polyphenolic substances, tannic acid (TA), a high-molecular-weight polyphenol composed of glucose as the core and multiple gallic acid groups connected by ester bonds, is rich in a large amount of catechin and epicatechin structures [28]. The presence of these groups not only enables TA to bind more stably to carbon materials through π-π interactions but also exerts stronger chelation and reduction effects on iron ions [29]. Based on this, the Cr(VI) adsorbents modified by TA have been increasingly studied by more and more researchers in recent years. For instance, Cheng et al. [30] achieved a Cr(VI) adsorption capacity of 179.22 mg/g using TA- modified graphene. Zhang et al. [31] demonstrated a 96.2% Cr(VI) removal efficiency by modifying ZVI with TA. Although the aforementioned studies have demonstrated the excellent adsorption performance of TA-modified carbon materials or TA-modified iron-based materials for Cr(VI), there is still a lack of comprehensive understanding regarding the synergistic effect of co-loading tannic acid and Fe_3_O_4_ on biochar for Cr(VI) removal.

In this study, tannic-acid/Fe_3_O_4_-modified corn straw biochar (Fe-TA-CSB) is prepared via a grinding-calcination method for the removal of Cr(VI) from acidic wastewater. Batch static adsorption experiments are conducted to investigate the effects of key factors such as the mass ratio of TA to Fe_3_O_4_, solution pH, adsorbent dosage, reaction time, initial Cr(VI) concentration, and coexisting ions on the Cr(VI) removal efficiency of Fe-TA-CSB. The adsorption characteristics of Fe-TA-CSB are analyzed based on kinetics, isotherms, and thermodynamic models. The physical-chemical properties of Fe-TA-CSB and the underlying Cr(VI) removal mechanism are analyzed using characterization techniques, including XRD, FTIR, XPS, and SEM. This study aims to solve the limitations of conventional materials for removing Cr(VI) under acidic conditions and provide insights into the design of stable iron-carbon composites for efficient Cr(VI) removal.

## 2. Result and Discussion

### 2.1. Characterization of Fe-TA-CSB

#### 2.1.1. XRD and FTIR Analysis

**Figure 1:**
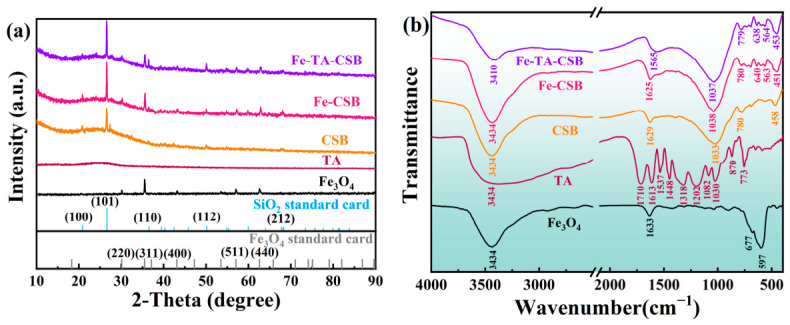
XRD (**a**) and FTIR (**b**) spectra of CSB, Fe-CSB, and Fe-TA-CSB.

Figure 1a shows the XRD patterns of the materials. For CSB, the peaks at 20.859°, 26.639°, 36.543°, 50.136°, and 67.742° are attributed to the characteristic peaks of the (100), (101), (110), (112), and (212) crystal planes of SiO_2_ (JCPDS 46-1045), indicating that CSB contains a relatively high amount of SiO_2_ [32]. For Fe-CSB and Fe-TA-CSB, the peaks of SiO_2_ present in pure CSB can still be observed, indicating that the crystal structure of the original biochar is not significantly altered by modification. In addition, new diffraction peaks at 30.12°, 35.48°, 43.12°, 57.03°, and 62.63° correspond to the (220), (311), (400), (511), and (440) crystal planes of the magnetite phase Fe_3_O_4_ (JCPDS 75-0033), showing that Fe_3_O_4_ is successfully coated on the materials [33]. Compared to Fe-CSB, the peak intensity of Fe_3_O_4_ is weakened in Fe-TA-CSB, indicating that TA and Fe may undergo a coordination reaction, reducing the crystallinity of their surfaces [34].

Figure 1b illustrates the FTIR spectra of Fe_3_O_4_, TA, CSB, Fe-CSB, and Fe-TA-CSB. Fe_3_O_4_ displays an O–H stretching vibrational peak at 3434 cm^−1^ and Fe–O bond peaks at 1633 cm^−1^, 677 cm^−1,^ and 597 cm^−1^ [35]. The characteristic peak at 1710 cm^−1^ is related to the C=O stretching vibration bands in TA [36], and the characteristic peaks at 1613 cm^−1^, 1537 cm^−1^, and 1448 cm^−1^ correspond to the vibration of the benzene ring skeleton, except for the O–H stretching vibration at 3434 cm^−1^ [37]. Meanwhile, the peaks at 1318 cm^−1^ and 1202 cm^−1^ are attributed to C–O bending vibration peaks [38], and peaks at 870 cm^−1^ and 773 cm^−1^ are the stretching vibration of C–H [39]. For CSB, the characteristic peak at 3434 cm^−1^ is related to the O–H stretching vibration peak, and the peak at 1629 cm^−1^ is attributed to the C=O or C=C stretching vibration in the aromatic group [40]. The peak at 1033 cm^−1^ belongs to C–O stretching vibration [41], and peaks at around 780 cm^−1^ and 460 cm^−1^ are attributed to O–Si–O stretching vibrations [42]. The weakened peak intensity of CSB and the new peaks at 563 cm^−1^ and 640 cm^−1^ corresponding to Fe–O bonds are observed in Fe-CSB, suggesting that Fe_3_O_4_ is successfully loaded onto the surface of Fe-CSB [43,44]. Compared to Fe-CSB, the spectrum of Fe-TA-CSB shows that the C=O/C=C peak shifts from 1625 cm^−1^ to 1565 cm^−1^, which is attributed to the coordination between Fe and TA molecules [45]. The O–H stretching vibration peak shifts from 3434 cm^−1^ to 3410 cm^−1^, which is related to the hydrogen bonding between TA and Fe-CSB, indicating the successful combination of TA with Fe-CSB.

In addition, the elemental analysis results (Table 1) show that the O/C ratio of CSB is 0.143, while the O/C ratios of Fe-CSB and Fe-TA-CSB increase to 0.300 and 0.232, respectively. This indicates that more oxygen-containing functional groups appear in biochar after modification, which further confirms the successful loading of Fe_3_O_4_ and TA onto the biochar [46].

#### 2.1.2. BET Analysis

**Figure 2:**
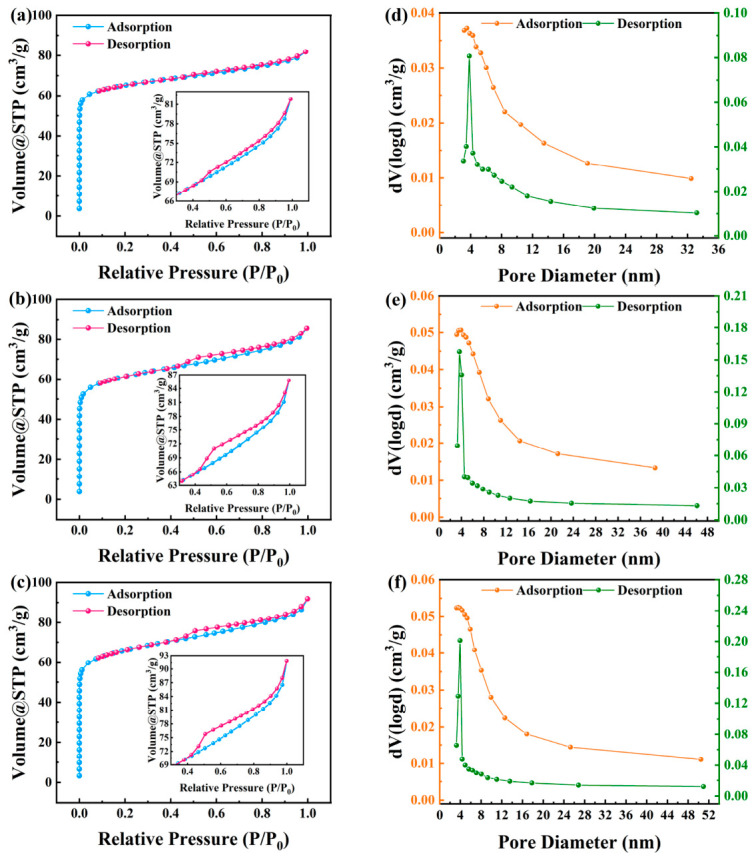
N_2_ adsorption–desorption isotherm curves of CSB (**a**), Fe-CSB (**b**), and Fe-TA-CSB (**c**); pore size distributions of CSB (**d**), Fe-CSB (**e**), and Fe-TA-CSB (**f**).

The adsorption–desorption isotherms and pore size distribution of materials under N_2_ conditions are shown in Figure 2 and Table 2. The results revealed that CSB, Fe-CSB, and Fe-TA-CSB exhibit type IV isotherms and show the hysteresis loop formed by capillary condensation in the range of higher relative pressure (p/p_0_ = 0.4–1.0) according to IUPAC classification [47]. Specifically, the adsorption curve is consistent with the desorption curve in relative pressure ranging from 0.1 to 0.4, indicating the presence of microporous structures [48]. However, the pore diameter of Cr(VI) species like ${\mathrm{HCrO}}_{4}^{-}$ and ${\mathrm{Cr}}_{2}O_{7}^{2-}$ is larger than that of micropores, making it difficult to diffuse into the internal structure of the micropores [49]. In contrast, the adsorption curve and the desorption curve begin to diverge in relative pressure ranging from 0.4 to 1.0, suggesting the presence of a large mesoporous structure [50]. This well-developed mesoporous structure provides rapid diffusion channels for Cr(VI), facilitating Cr(VI) access to internal active sites for adsorption and reduction [51]. The results seen in Table 2 show the BET surface area, total pore volume, and pore diameter. Compared to CSB, the average pore size of Fe-CSB increased from 1.996 to 2.29 nm, while the specific surface area (S_BET_) decreased from 253.604 to 231.774 m^2^/g. This change is due to the fact that the mesoporous structure remains intact, whereas partial micropores are obstructed by Fe_3_O_4_ particles, ultimately reducing the specific surface area of biochar [52]. In contrast, the specific surface area and the average pore size of Fe-TA-CSB were 250.37 m^2^/g and 2.27 nm. This suggests that the coordination of TA with Fe_3_O_4_ could effectively prevent the agglomeration of iron nanoparticles and make them evenly distributed on the surface of the material, thereby solving the problem of pore blockage [53].

#### 2.1.3. TG-DTG and VSM

**Figure 3:**
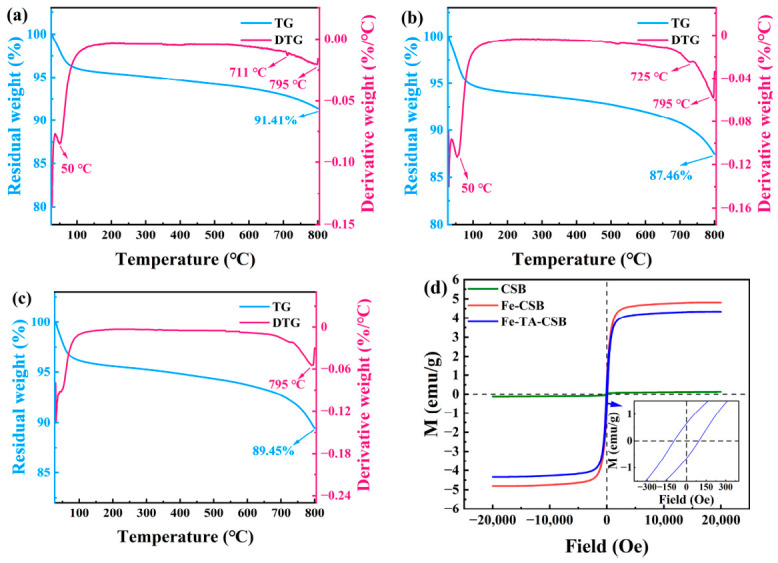
The TG-DTG curves of CSB (**a**), Fe-CSB (**b**), and Fe-TA-CSB (**c**); the magnetic hysteresis loop of Fe-TA-CSB (**d**).

The TGA-DTG curves of CSB, Fe-CSB, and Fe-TA-CSB are shown in Figure 3a–c. The figures reveal two mass loss stages of the three materials. In the first stage, the mass loss below 100 °C belongs to the evaporation of water [54]. In the second stage, the mass loss between 100 °C and 800 °C is assigned to the gradual decomposition of lignin over a wide temperature interval, including the process of strong cracking below 700 °C and the process of charring reactions above 700 °C [55]. As the decomposition of lignin extends to a temperature of 800 °C, the amounts of solid residue of the three materials are above 85%, suggesting that these materials exhibit high heat stability. This is due to lignin, as a polymeric aromatic compound, which has a low amount of mass loss and a low decomposition rate and can convert into more stable structures at high temperatures [56].

The hysteresis loops of materials by the VSM test are shown in Figure 3d. The saturation magnetization value of CSB is 0.122 emu/g, indicating that raw CSB is non-magnetic. Compared to CSB, the saturation magnetization values of Fe-CSB and Fe-TA-CSB are 4.81 and 4.33 emu/g, indicating that the CSB materials after modification have sufficient magnetic properties, facilitating magnetic separation [57,58]. The decrease from Fe-CSB to Fe-TA-CSB is attributed to the loading of non-magnetic TA, while this change has no adverse effect on their magnetic separation ability. The VSM results suggest that Fe-TA-CSB can successfully separate from water after adsorption under the action of a magnetic field.

### 2.2. Influencing Factors

#### 2.2.1. Effect of TA/Fe_3_O_4_ Mass Ratio

**Figure 4:**
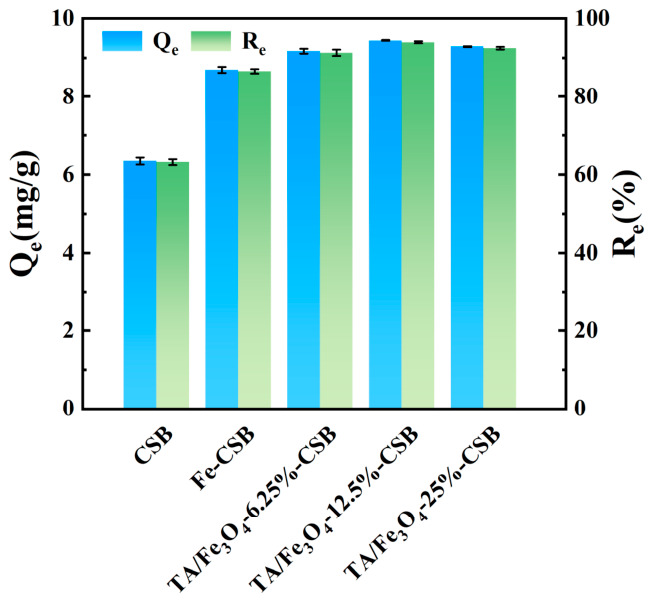
Adsorption capacity (*Q_e_*) and removal efficiency (*R_e_*).

To investigate the effect of CSB materials loaded with different TA/Fe_3_O_4_ mass ratios on the adsorption of Cr(VI), the contents of CSB and Fe_3_O_4_ were fixed at 3 g and 0.225 g, and different masses of TA were added according to TA/Fe_3_O_4_ mass ratios of 6.25%, 12.5%, and 25%. These composites were prepared for static adsorption experiments to choose those with the best Cr(VI) removal performance. As shown in Figure 4, the equilibrium adsorption capacity (*Q_e_*) and the removal efficiency (*R_e_*) of Cr(VI) by CSB were 6.35 mg/g and 63.22%, respectively. Compared to pristine CSB, the *Q_e_* and *R_e_* values of Fe-CSB increased to 8.69 mg/g and 86.53%, with the increases of 2.34 mg/g and 23.31%, respectively. Additionally, both *Q_e_* and *R_e_* values of CSB modified by TA/Fe_3_O_4_ were improved to various degrees. Among them, the order of *Q_e_* and *R_e_* values from the highest to the lowest were: TA/Fe_3_O_4_-12.5%-CSB (9.44 mg/g and 94.02%), TA/Fe_3_O_4_-25%-CSB (9.29 mg/g and 92.48%), and TA/Fe_3_O_4_-6.25%-CSB (9.17 mg/g and 91.30%), respectively. This indicates that the co-modification with TA and Fe_3_O_4_ effectively enhances the adsorption capacity of Cr(VI) by CSB. The *Q_e_* and *R_e_* values decreased when TA was added beyond the TA/Fe_3_O_4_ ratio of 12.5%, which could be attributed to the agglomeration of excessive tannic acid, covering the active sites on the surface of the material. Therefore, the TA/Fe_3_O_4_ modified CSB composite with a TA/Fe_3_O_4_ mass ratio of 12.5% (0.028 g TA, 0.225 g Fe_3_O_4,_ and 3 g CSB) is selected for subsequent experimental studies and named as Fe-TA-CSB, considering the adsorption capacity and removal efficiency of Cr(VI).

#### 2.2.2. Effect of pH

**Figure 5:**
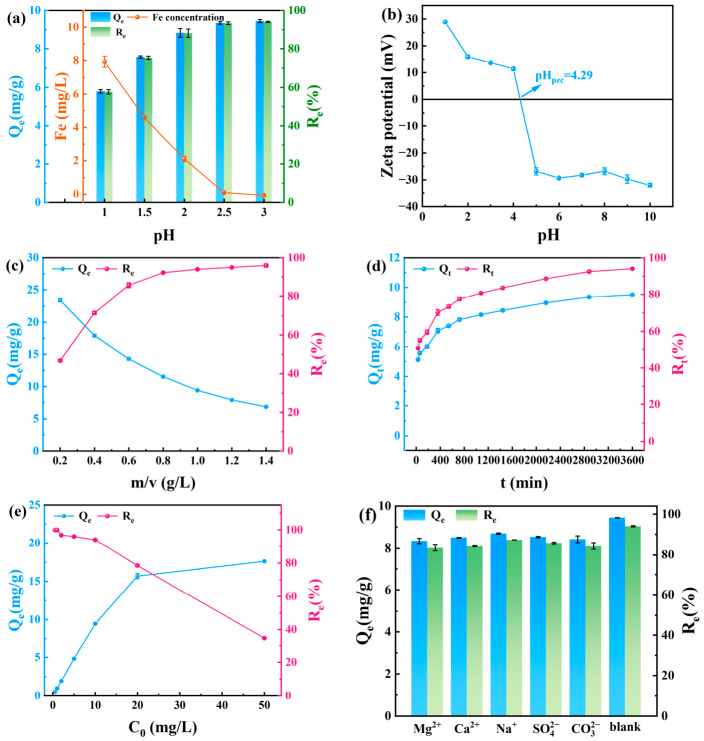
Effects of solution pH (**a**), zeta potential (**b**), solid–liquid ratio (**c**), adsorption time (**d**), initial Cr(VI) concentration (**e**), and coexisting ions (**f**) on the removal of Cr(VI) by Fe-TA-CSB.

Considering that actual industrial wastewater and acidic mine wastewater typically exhibit extremely acidic conditions with pH values below 3, the effect of pH on the removal of Cr(VI) was investigated by choosing pH values from 1 to 3 [59,60]. As the initial pH of the solution increased from 1.0 to 3.0, the removal efficiency (*R_e_*) gradually increased from 57.71% to 94.02%, and the equilibrium adsorption capacity (*Q_e_*) increased from 5.79 mg/g to 9.45 mg/g. This trend can be attributed to the speciation of Cr(VI) and the surface charge properties of the adsorbent. Within the pH range of 1.0–3.0, Cr(VI) predominantly exists as negatively charged species, including ${\mathrm{HCrO}}_{4}^{-}$ and ${\mathrm{Cr}}_{2}O_{7}^{2-}$ [61]. As shown in Figure 5b, the pH value at the point of zero charges (pH_pzc_) for Fe-TA-CSB is 4.29, indicating that the surface of the Fe-TA-CSB is positively charged when the pH value is below 4.29, making it favorable for electrostatic attraction between the adsorbent and the anionic Cr(VI) species [62]. When pH values are lower than 3, the decline in removal efficiency below this point is attributed to the fact that protons (H^+^) with high concentration suppress Cr(VI) adsorption by the competition with Cr(VI) species for the limited active binding sites [63].

Furthermore, the chemical stability of the adsorbent, particularly the leaching of iron from the composite under strongly acidic conditions, represents another critical factor that must be evaluated for the selection of an optimal pH value. The concentrations of Fe over a pH change of 1.0–2.0 were above 2.1 mg/L after Cr(VI) adsorption, which may be due to the decomposition of iron oxides on the surface of Fe-TA-CSB, resulting in a decrease in the active sites of the adsorbent [64]. In contrast, the dissolution of Fe was 0.106 mg/L and 0 mg/L when pH = 2.5 and 3.0, respectively, lower than the WHO drinking water Fe limit of 0.3 mg/L. Considering the best removal efficiency and the solubility of Fe in the aqueous solution, subsequent experiments were carried out at pH = 3.

#### 2.2.3. Effect of Solid-to-Liquid Ratio (*m*/*v*)

To investigate the effect of solid-to-liquid ratio (*m*/*v*) on the adsorption of Cr(VI) by Fe-TA-CSB, static adsorption experiments were conducted with various masses of the Fe-TA-CSB material and the fixed volume of the Cr(VI) solution at ratios of 0.2, 0.4, 0.6, 0.8, 1.0, 1.2, and 1.4 g/L. As shown in Figure 5c, the removal efficiency (*R_e_*) increased rapidly from 46.78% to 94.00% as the *m*/*v* increased from 0.2 to 1.0 g/L, while the equilibrium adsorption capacity (*Q_e_*) decreased from 23.43 mg/g to 9.41 mg/g. This is because the increase in *m*/*v* represents the increase in the dose of adsorbent, while the initial Cr(VI) concentration remains unchanged. This means that more adsorption sites are available on the material to contact with Cr(VI) as the *m*/*v* increases, leading to a continuous rise in the removal rate and a continuous decrease in the equilibrium adsorption capacity [65]. However, when the *m*/*v* exceeded 1.0 g/L, the *R_e_* of Cr(VI) increased slowly to 95.97%, while the *Q_e_* decreased gradually to only 6.87 mg/g, indicating that excessive *m*/*v* conditions may result in a large number of adsorption sites remaining unsaturated after the adsorption of Cr(VI) [66]. Considering the *Q_e_* values and the *R_e_* values, *m*/*v* = 1.0 g/L was selected for subsequent experiments.

#### 2.2.4. Effect of Contact Time

To investigate the effect of contact time on Cr(VI) removal, adsorption experiments were conducted at contact times of 30, 60, 180, 360, 540, 720, 1080, 1440, 2160, 2880, and 3600 min, respectively. As shown in Figure 5d, the removal efficiency (*R_e_*) of Cr(VI) increased from 50.72% to 94.05% with the increase in the contact time, and the equilibrium adsorption capacity (*Q_e_*) increased from 5.13 mg/g to 9.50 mg/g. The whole adsorption process can be divided into three stages. Specifically, during the initial adsorption stage (0–360 min), a large number of unsaturated adsorption sites exist on the surface of the adsorbent, leading to a rapid increase in the adsorption amount and removal rate. During the middle stage of adsorption (540–1440 min), the adsorption sites on the material surface are gradually occupied, making the growth rates of both *R_e_* and *Q_e_* slower compared to the previous period. In the late adsorption stage (2160–3600 min), the active adsorption sites on the surface of Fe-TA-CSB become saturated, leading to the saturation of adsorption [67]. Based on the above analysis, the adsorption equilibrium duration of Fe-TA-CSB for Cr(VI) is approximately 3600 min, and the equilibrium adsorption rate exceeds 94%. However, it is noted that 3600 min is relatively long for practical wastewater treatment applications. Similar slow kinetics have been reported for other Fe-based biochar adsorbents. For instance, Liu et al. [68] developed magnetic biochar derived from peanut hull for Cr(VI) removal, which required approximately 60 h to reach equilibrium with an adsorption capacity of only 6.64 mg/g at pH 6.0. Tang et al. [69] synthesized a novel pyrite/biochar composite (BM-FeS_2_@BC700) by ball milling technology and reported an enhanced Cr(VI) removal capacity of 134 mg/g, but the equilibrium time extended to 72 h (4320 min). This slow kinetics behavior is primarily because, under the conditions of internal pressure and water surface tension, Cr(VI) cannot access iron oxide particles distributed inside the bottleneck pores of materials, requiring dozens of hours or even a week to reach adsorption equilibrium [70]. This indicated that the effect of contact time on Cr(VI) is regulated by multiple factors, such as the number of active sites, the driving force for Cr(VI) to enter the pores of the material, and the mass transfer resistance inside the pores [71].

#### 2.2.5. Effect of Initial Concentration

The adsorption performance of Cr(VI) by Fe-TA-CSB under the initial Cr(VI) concentrations (*C*_0_ = 0.5–50 mg/L) is shown in Figure 5e. The figure shows that the removal efficiency (*R_e_*) and the equilibrium adsorption capacity (*Q_e_*) show a significant and differentiated changing trend with the gradual increase in the initial Cr(VI) concentration. When *C*_0_ increased from 0.5 to 10 mg/L, the *R_e_* values gradually decreased from 100.00% to 94.02%, while the *Q_e_* values rapidly increased from 0.5 mg/g to 9.44 mg/g. The high *R_e_* value (>94%) at this range may be due to sufficient active adsorption sites on the surface of the Fe-TA-CSB relative to the amount of Cr(VI) in the solution at low concentrations, thereby enabling most of the Cr(VI) to be efficiently adsorbed. However, the value of *R_e_* rapidly decreased from 78.47% to 34.77% with the increasing *C*_0_ from 20 mg/L to 50 mg/L, and the *Q_e_* values rapidly increased from 15.70 mg/g to 17.62 mg/g. This may be because the dosage of the Fe-TA-CSB is fixed, which means the number of active sites on its surface remains unchanged. With the continuous increase in the initial concentration, adsorption sites become rapidly saturated, leaving most of the Cr(VI) unadsorbed and causing the adsorption efficiency to decrease rapidly [72].

#### 2.2.6. Effect of Coexisting Ions

Figure 5f shows the effect of various cations and anions (at the concentration of 10 mM) on Cr(VI) removal by Fe-TA-CSB under the condition of *C*_0_ = 10 mg/L and pH = 3.0. The results show that ${\mathrm{Mg}}^{2+}$, ${\mathrm{Ca}}^{2+}$, ${\mathrm{Na}}^{+}$, ${\mathrm{CO}}_{3}^{2-}$, and ${\mathrm{SO}}_{4}^{2-}$ have suppressive effects on the removal of Cr(VI) compared with the blank group without interfering ions (*R_e_* = 94.02%), and the removal efficiency (*R_e_*) values decreased to 83.52%, 84.39%, 87.27%, 85.69%, and 84.35%, respectively. Among them, ${\mathrm{Na}}^{+}$ has the least effect on the adsorption of Cr(VI), which is because the Na^+^ ion redox potential is lower than that of Cr(VI). In contrast, ${\mathrm{Mg}}^{2+}$ and ${\mathrm{Ca}}^{2+}$ exhibit stronger inhibitory effects on Cr(VI) adsorption than ${\mathrm{Na}}^{+}$. This is attributed to the generation of films on the surface of Fe-TA-CSB through the interaction between ${\mathrm{Mg}}^{2+}/{\mathrm{Ca}}^{2+}$ and the oxygen-containing group of the adsorbent, thus preventing the contact of Cr(VI) and active sites [73]. The reduction of Cr(VI) adsorption by adding ${\mathrm{CO}}_{3}^{2-}$ is due to the formation of carbonate-Fe compounds (FeCO_3_) through a reaction between ${\mathrm{CO}}_{3}^{2-}$ and iron, resulting in hindering the Cr(VI) removal by blocking active sites on the surface of the adsorbent. The presence of ${\mathrm{SO}}_{4}^{2-}$, leading to the decrease in the *R_e_* value of Cr(VI), is related to its competitive adsorption with dichromate ions at surface binding sites [74].

### 2.3. Adsorption Characteristics

#### 2.3.1. Adsorption Kinetics

**Figure 6:**
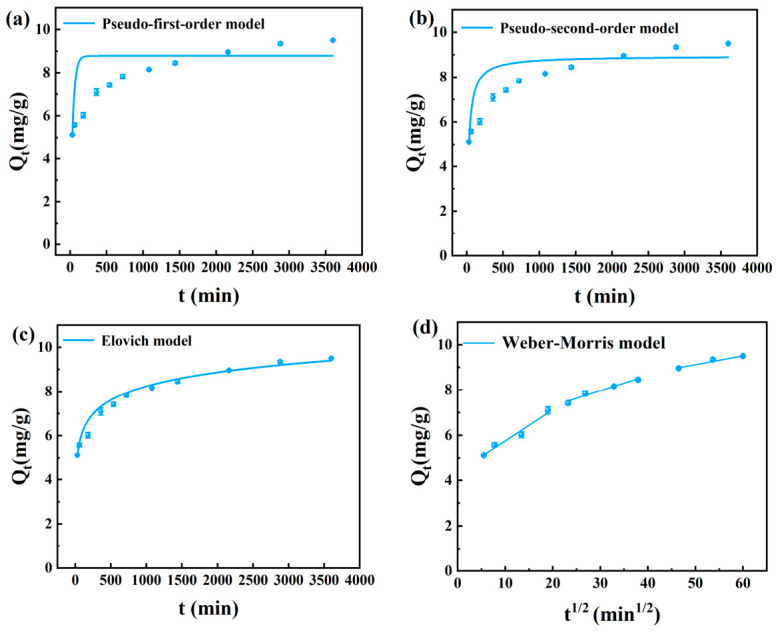
Fitting of the adsorption kinetics model of Cr(VI) adsorption: (**a**) pseudo-first-order; (**b**) pseudo-second-order; (**c**) Elovich; and (**d**) the Weber–Morris model.

The experimental data were fitted by the pseudo-first-order, pseudo-second-order, and Elovich kinetic models in order to study the adsorption rate and adsorption behavior of Cr(VI) by Fe-TA-CSB during the adsorption process. As shown in Figure 6a–c and Table 3, the coefficient of determination (*R*^2^ ) of the Elovich kinetic model is the highest ( *R*^2^ > 0.99) among the three models, indicating that the adsorption process of Fe-TA-CSB in this study was a heterogeneous diffusion adsorption dominated by chemical adsorption [75]. The experimental data were further fitted by the Weber–Morris kinetic model. As shown in Figure 6d, the adsorption process of Cr(VI) includes the rapid diffusion of Cr(VI) on the surface of Fe-TA-CSB (0–360 min), the slow diffusion into the pore (540–1440 min), and reaction equilibrium (2160–3600 min) [76]. In addition, *Q_t_* and *t*^1/2^ in the graph exhibit a linear relationship, but these related lines do not pass through the origin, indicating that the adsorption of Cr(VI) by Fe-TA-CSB involves multiple mechanisms, and the intraparticle diffusion mechanism is not the only control pathway. 

#### 2.3.2. Adsorption Isotherms

**Figure 7:**
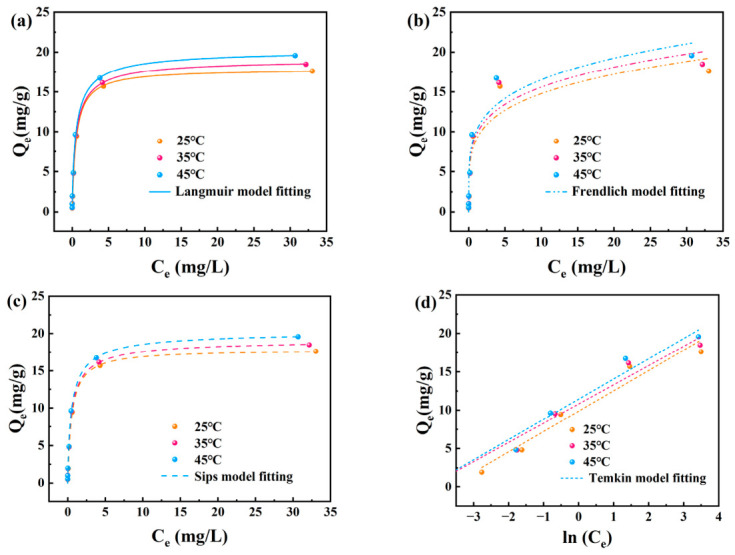
Adsorption isotherm fitting curves of Cr(VI) on composite: (**a**) Langmuir; (**b**) Freundlich; (**c**) Sips; and (**d**) Temkin.

Figure 7 and Table 4 show the fitting parameters and results for Cr(VI) adsorption using the Langmuir, Freundlich, Sips, and Temkin models at different temperatures. Among them, the Langmuir model is commonly used to describe monolayer chemisorption behavior; the Freundlich model is commonly used to describe multilayer physical adsorption behavior on heterogeneous surfaces [77]; and the Sips model is a combination of the Langmuir and the Freundlich models, and the parameter m in the Sips equation can be used to evaluate the homogeneity of the adsorbent surface [78]. The Temkin adsorption isotherm model is used to describe the interactions between the adsorbent and adsorbate molecules [79]. The results show that the coefficients of determination (*R*^2^) of the Freundlich model are lowest among the four models, indicating that the process of adsorption is not dominated by multilayer physical adsorption. In contrast, the high coefficients of determination (*R*^2^ > 0.99) of Langmuir and Sips indicate that the adsorption process was primarily associated with monolayer chemisorption. Nevertheless, further evaluation of the material’s heterogeneity was required through analysis of the Sips model exponent. The *m* values of the Sips model are less than 1 at 25, 35, and 45 °C, showing that the surface of the adsorbent is heterogeneous [80]. In general, the Sips isotherm model is the most suitable to describe the adsorption process, and the *Q_m_* values of Cr(VI) fitted by the Sips model are 17.89, 19.18, and 20.35 mg/g under the temperatures of 25 °C, 35 °C, and 45 °C, respectively, which are close to the experimental results.

#### 2.3.3. Adsorption Thermodynamics

**Figure 8:**
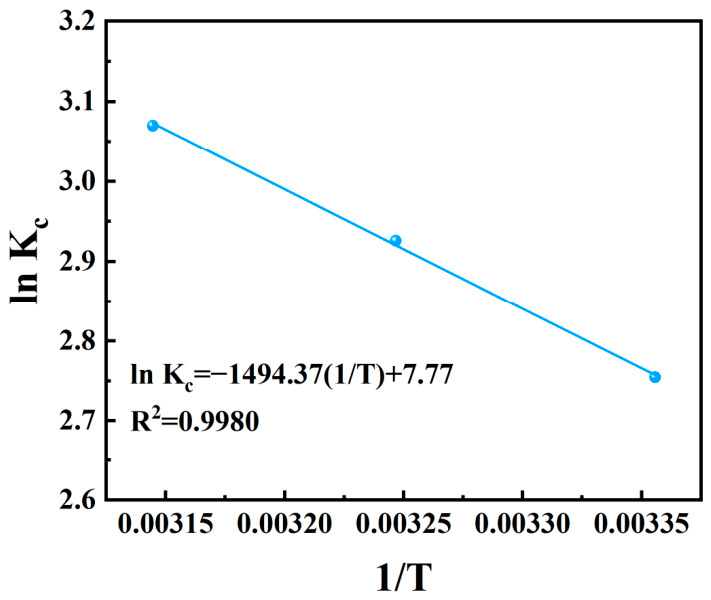
Adsorption thermodynamics curve for Cr(VI) adsorption.

The thermodynamic curve and thermodynamic parameters of Cr(VI) by Fe-TA-CSB are shown in Figure 8 and Table 5. It can be seen that $\Delta H^{\theta }\;$= 12.42 kJ/mol is positive, indicating that adsorption is an endothermic reaction. The positive $\Delta S^{\theta }$ value of 64.62 J/(mol·K) reveals that the Cr(VI) adsorption is a large degree of disorder or randomness in the system [81]. The $\Delta G^{\theta }$ values are negative at −6.825 kJ/mol (298 K), −7.492 kJ/mol (308 K), and −8.116 kJ/mol (318 K), and the absolute values of $\Delta G^{\theta }$ increase with increasing temperature. This indicates that the adsorption process of Cr(VI) is spontaneous and Cr(VI) removal efficiency is improved at a higher temperature [82].

### 2.4. Renewability and Performance Evaluation

**Figure 9:**
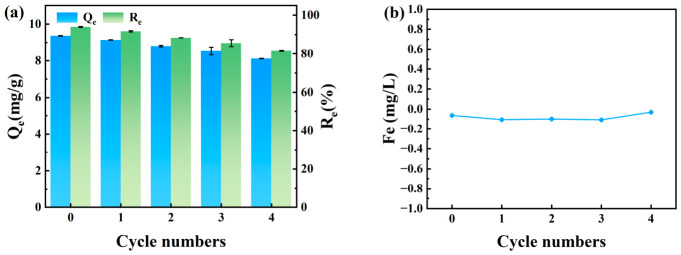
(**a**) Adsorption capacity (*Q_e_*) and removal efficiency (*R_e_*) of Cr(VI) and (**b**) Fe leaching concentrations over four times of regeneration cycles.

Figure 9 shows the changes in the removal efficiency (*R_e_*) and adsorption capacity (*Q_e_*) of Cr(VI) by Fe-TA-CSB after desorption using 0.1 M HCl under the conditions of concentration of Cr(VI) of 10 mg/L and pH = 3. It can be seen that the *R_e_* and *Q_e_* values exhibit a decreasing trend after desorption. Specifically, after four experimental cycles, the equilibrium adsorption capacity (*Q_e_*) and the removal efficiency (*R_e_*) decreased to 8.12 mg/g and 81.44%, respectively. This is due to the gradual blocking and depletion of active sites on the surface of the adsorbent. However, the Fe leaching during the adsorption–desorption process is below the detection limit, indicating that Fe-TA-CSB has no risk of causing secondary iron contamination despite the gradual depletion of active sites during desorption. This result is inconsistent with the previously reported result that some iron-based biochar materials are prone to oxidation and leach iron ions during desorption [83]. Combined with the maintained Cr(VI) removal efficiency (>80%) after four cycles, Fe-TA-CSB shows a possibility for repeated use.

Table 6 illustrates the variation in hexavalent chromium adsorption capacity of different magnetic biochars at low pH values. In this study, Fe-TA-CSB achieved a maximum equilibrium adsorption capacity of 17.89 mg/g for Cr(VI) at initial concentrations ranging from 0.5 to 50 mg/L. In addition, the iron ion leaching was not observed at pH = 3.0, and the removal rate remains above 80% after four cycles during the reproductive experiment. However, within the same Cr(VI) concentration range, some traditional iron-based biochar materials listed in the table not only exhibited lower maximum adsorption capacities compared to the material developed in this study but also lacked evaluation of iron leaching and regeneration performance, such as TP-SSA (2.52 mg/g) [84], MHDB (9.95 mg/g) [85], m-biochar (9.92 mg/g) [86], GSMB (9.11 mg/g) [87] and nZVI-biochar (13.27 mg/g) [88]. This proves that the material in this study overcomes the critical drawbacks of iron leaching and poor regeneration commonly found in traditional iron-based biochar. Nevertheless, a limitation of this study is that the performance of Fe-TA-CSB for Cr(VI) at higher concentration conditions remains to be further evaluated, compared to the high-capacity adsorbents at the highest concentration of 500 mg/L, such as BMBC (66.10 mg/g) [89] and MFBAP (80.58 mg/g) [90].

### 2.5. Removal Mechanism of Cr(VI) by Fe-TA-CSB

**Figure 10:**
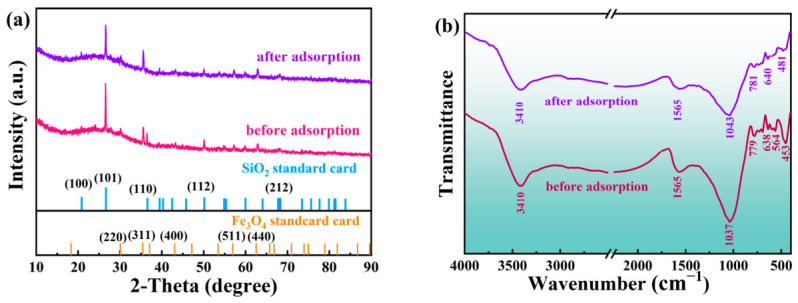
XRD (**a**) and FTIR (**b**) before and after Cr(VI) adsorption by Fe-TA-CSB.

Figure 10a shows the XRD pattern of Fe-TA-CSB before and after adsorbing Cr(VI). After the adsorption of Cr(VI), the intensity of the characteristic peak of SiO_2_ decreases at 26.639° and 36.543°, indicating that Cr(VI) species are adsorbed onto the material [91]. However, no significant change was observed in the characteristic peak of Fe_3_O_4_. This may be due to the structural stability of Fe_3_O_4_ in the composite, while the adsorbed Cr(VI) exerts only a slight effect on the Fe_3_O_4_ structure [92].

Figure 10b shows the FTIR spectra of Fe-TA-CSB before and after the adsorption of Cr(VI). The characteristic vibration of the C=O/C=C peak at 1565 cm^−1^ becomes broadened [93]. The characteristic vibration for the C–O bond in the aromatic ring shifts from 1037 cm^−1^ to 1043 cm^−1^ [94]. This indicates the participation of these functional groups in the adsorption of Cr(VI). In addition, a new characteristic peak appears at 480 cm^−1^ after adsorption. This may be attributed to the formation of a new complex of Cr–O, indicating the successful reduction in Cr(VI) to Cr(III) and immobilization in the form of oxide on the surface of Fe-TA-CSB [76].

**Figure 11:**
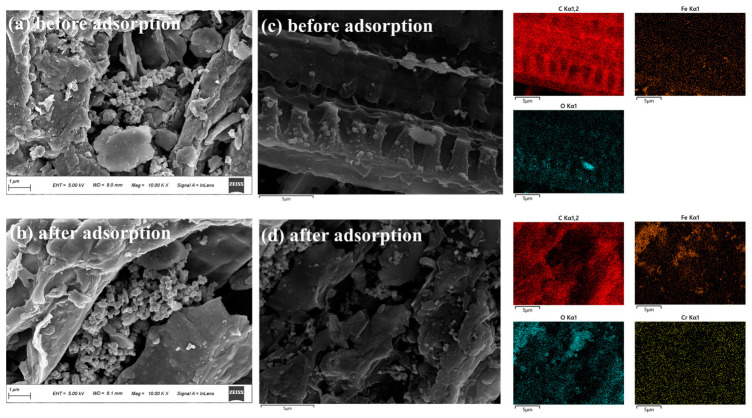
SEM-images of Fe-TA-CSB before (**a**) and after (**b**) adsorption of Cr(VI); mapping patterns before (**c**) and after (**d**) adsorption of Cr(VI).

Figure 11a,b show the SEM-images of Fe-TA-CSB before and after adsorbing Cr(VI). As shown in Figure 11a, a large amount of irregular pores exist on the surface of Fe-TA-CSB, indicating that the adsorbent could provide effective adsorption sites for Cr(VI) removal [95]. Meanwhile, the presence of many agglomerate-like spherical particles is attributed to the formation of Fe_3_O_4_-TA complexes, which is consistent with the findings of Gopi et al. [96]. After the adsorption of Cr(VI), the surface of Fe-TA-CSB shows rough features, and the aggregation of particles increases (Figure 11b). This is possibly because the adsorbent combined with Cr(VI) ions through physical and chemical interaction, leading to the formation of stable chromium-containing complexes accumulating on the pore surfaces [97]. The SEM-mapping images of Fe-TA-CSB before and after Cr(VI) adsorption are presented in Figure 11c,d. It can be seen that C, O, and Fe are uniformly distributed across the surface of the Fe-TA-CSB (Figure 11c). After adsorption, these elements are still present, while a new element of Cr appears and exhibits homogeneous distribution on the Fe-TA-CSB surface (Figure 11d), further suggesting that Cr(VI) has been successfully adsorbed onto the Fe-TA-CSB surface [98].

**Figure 12:**
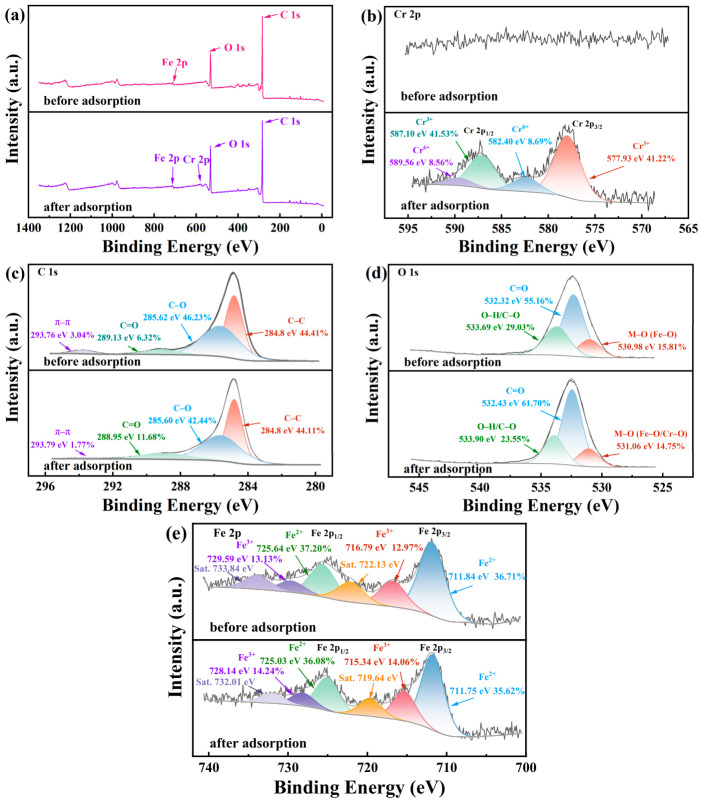
XPS full spectra (**a**) and single element spectra of Cr 2p (**b**), C 1s (**c**), O 1s (**d**), and Fe 2p (**e**) for Fe-TA-CSB before and after adsorption.

Figure 12 shows the XPS scanning results of Fe-TA-CSB before and after Cr(VI) adsorption. The peaks at 285.12 eV, 532.38 eV, and 711.92 eV are observed in the full spectra (Figure 12a), corresponding to C 1s, O 1s, and Fe 2p, respectively. After adsorption, a new energy peak appearing at 577.91 eV is attributed to Cr 2p, indicating that Cr(VI) has been successfully adsorbed to the surface of Fe-TA-CSB.

According to the spectra of Cr 2p (Figure 12b), the energy peaks at 577.93 eV (Cr 2p_3/2_) and 587.10 eV (Cr 2p_1/2_) are assigned to Cr(III), and the peaks at 582.40 eV (Cr 2p_3/2_) and 589.56 eV (Cr 2p_1/2_) are attributed to Cr(VI). Among them, the relative content of Cr(III) (82.75%) is higher than that of Cr(VI) (17.25%), indicating that the adsorbed Cr(VI) has been partially reduced to Cr(III) during the removal of Cr(VI) by Fe-TA-CSB [99].

Figure 12c shows the C 1s spectra of Fe-TA-CSB before and after Cr(VI) adsorption. The binding energy peaks at 284.8 eV, 285.62 eV, 289.13 eV, and 293.76 eV are C=C, C–O, C=O, and π–π* bonding, respectively [100,101,102]. After adsorption, the relative content of C–O decreased from 46.23% to 42.33%, while that of C=O increased from 6.32% to 11.68%. This may be because the C–O group with the function of electron donor participates in the reduction in Cr(VI) to Cr(III), and is oxidized to C=O group [103].

From the O 1s spectra in Figure 12d, the signal peak at 530.98 eV is related to the metal oxide bond (M–O), probably corresponding to the Fe–O bond in this study. The peaks at 532.32 eV and 533.69 eV are O–H/C–O and C=O, respectively [104]. After adsorption, the binding energy position of M–O shifted from 530.98 eV to 531.06 eV. This was inferred to be associated with the formation of a new metal-based oxide bond, possibly involving the Cr–O bond [105]. Meanwhile, the relative content of C=O increased from 55.16% to 61.70%, and the relative content of O–H/C–O decreased from 29.03% to 23.55%. The relative content of M–O decreased from 15.81% to 14.75%. This suggests that the form of the Cr–O bond is related to the complexation of reduced Cr(III) with oxygen functional groups like Fe–O, C–O, and O–H.

Figure 12e shows the Fe 2p spectra before and after Cr(VI) adsorption. It can be seen that the peaks at 711.84 eV and 725.64 eV are attributed to Fe 2p_3/2_ and Fe 2p_1/2_ of Fe(II), and the peaks at 716.79 eV and 729.59 eV correspond to Fe 2p_3/2_ and Fe 2p_1/2_ of Fe(III). In addition, the peaks of the satellites of Fe 2p are observed at 722.13 eV and 733.84 eV, respectively. After adsorption, the total relative content of Fe(III) increased from 26.10% to 28.30%, and the total relative content of Fe(II) reduced from 73.91% to 71.70%. This may be due to the fact that Fe(II) is involved in Cr(VI) reduction through electron transfer and is oxidized to Fe(III) [106].

**Figure 13:**
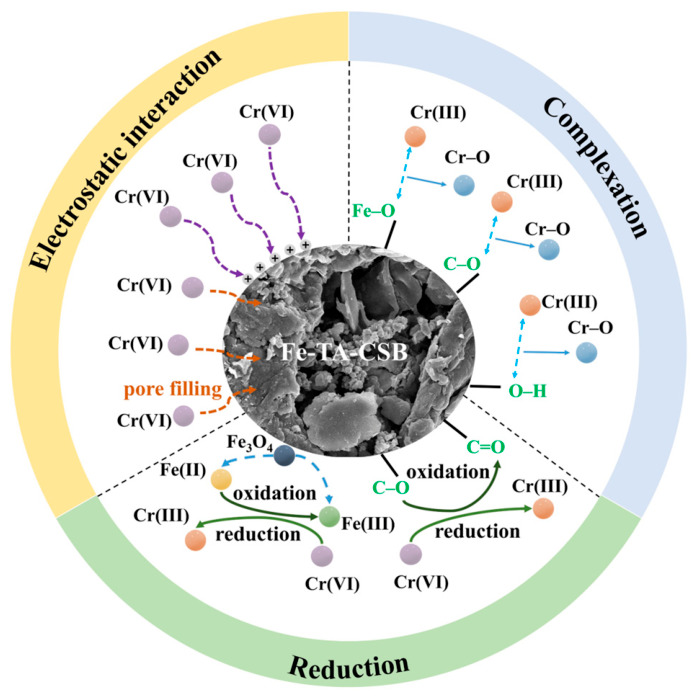
The proposed mechanism of Cr(VI) removal by Fe-TA-CSB.

In summary, the removal mechanism of Cr(VI) in acidic wastewater by the Fe-TA-CSB composite mainly includes electrostatic interaction, reduction, and complexation. The specific reaction process is shown in Figure 13. Cr(VI) is trapped to the surface of Fe-TA-CSB through electrostatic attraction with the positively charged surface. Cr(VI) is adsorbed through pore filling due to the large surface area and rich mesoporous structure of Fe-TA-CSB. Adsorbed Cr(VI) is reduced to Cr(III) through electron transfer of C–O and Fe(II). Reduced Cr(III) is complexed with oxygen functional groups such as Fe–O, O–H, and C–O in the formation of Cr–O immobilizing on the surface of Fe-TA-CSB.

## 3. Materials and Methods

### 3.1. Materials and Reagents

The chemicals: potassium dichromate (K_2_CrO_7_), sodium hydroxide (NaOH), and hydrochloric acid (HCl) were all analytical grade and purchased from Xilong Technology Co., Ltd. (Shanghai, China). Corn straw was purchased from Lize Environmental Protection Technology Co., Ltd. (Zhengzhou, China). Nanoscale Fe_3_O_4_ (analytically pure, 99.99%, black powder) was purchased from Hebei Yuanying New Materials Co., Ltd. (Xingtai, China). Tannic acid (analytically pure, 98%, tan powder) was purchased from Shanghai Macklin Biochemical Co., Ltd. (Shanghai, China). Deionized water (18.2 MΩ∙cm) was prepared by the Milli-Q water system (Millipore, Burlington, MA, USA). A 1000 mg/L stock solution of Cr(VI) was prepared by dissolving K_2_CrO_7_ in deionized water and was then diluted to the required concentration for batch experiments.

### 3.2. Synthesis of Adsorbents

#### 3.2.1. Preparation of CSB

CSB was produced from corn straw via pyrolysis under oxygen-limited conditions. The corn straw was cleaned and dried, then sieved through a 100-mesh screen to obtain corn stalk powder. The powder was placed in a covered ceramic crucible and heated in a muffle furnace to 700 °C at a rate of 10 °C/min, then maintained at this temperature for 120 min to allow complete carbonization. After cooling naturally to room temperature inside the furnace, the resulting biochar powder was washed with a large amount of deionized water until neutral (pH = 7.0), then dried in an oven at 60 °C. After drying, it was stored in a sealed container for later use. The obtained biochar was named CSB.

#### 3.2.2. Preparation of Fe-TA-CSB

Fe-TA-CSB was mainly prepared by mixing CSB with Fe_3_O_4_ and TA, followed by secondary pyrolysis. Specifically, 3 g of CSB, 0.225 g of nano-sized Fe_3_O_4_ powder, and 0.028 g of tannic acid (m(TA):m(Fe_3_O_4_) = 12.5%, which was determined by Section 2.2.1) were added to an agate mortar and thoroughly ground to achieve a homogeneous mixture. Subsequently, the mixture was pyrolyzed, washed, and dried under the same conditions as the preparation process of CSB, and the obtained product was named Fe-TA-CSB. Meanwhile, a Fe_3_O_4_ modified biochar without TA was prepared by repeating the above steps, and was named as Fe-CSB.

### 3.3. Adsorption Experiments

0.05 g of Fe-TA-CSB was added to 50 mL 10 mg/L Cr(VI) solution. After 60 h of reaction at 25 °C and pH 3.0, the supernatant was collected to determine the Cr(VI) concentration. Batch adsorption experiments were conducted to further explore the effects of pH (1.0–3.0), mass-to-volume ratio (*m*/*v*) (0.2–1.4 g/L), initial Cr(VI) concentration (0.5–50 mg/L), contact time (30–3600 min), temperature (25 °C, 35 °C, 45 °C), and coexisting ions (${\mathrm{Mg}}^{2+}$, ${\mathrm{Ca}}^{2+}$, ${\mathrm{Na}}^{+}$, ${\mathrm{CO}}_{3}^{2-}$, and ${\mathrm{SO}}_{4}^{2-}$) on adsorption of Cr(VI). The pH was adjusted by 0.1 mol/L HCl and NaOH. On this basis, kinetic, isothermal, and thermodynamic experiments were carried out at the optimum reaction conditions of pH and *m*/*v*. The above experiments were conducted in triplicate in a constant temperature water bath shaker at 150 rpm. The removal efficiency of Cr(VI) (*R_e_*, %) and equilibrium adsorption (*Q_e_*, mg/g) were calculated using the following Equations (1) and (2).

(1) $$R_{e}=\frac{C_{0}-C_{e}}{C_{0}}$$

(2) $$Q_{e}=\frac{C_{0}-C_{e}}{m}\times V$$

where *R_e_* is the removal efficiency (%) of the material for Cr(VI) at adsorption equilibrium, *C*_0_ is the initial concentration of Cr(VI) (mg/L), *C_e_* is the concentration of Cr(VI) at adsorption equilibrium (mg/L), *Q_e_* is the equilibrium adsorption capacity (mg/g), *V* is the volume of the solution containing Cr(VI) (L), and *m* is the mass of the adsorbent (g).

### 3.4. Adsorption Kinetics, Isotherms, and Thermodynamics Models

#### 3.4.1. Adsorption Kinetics Model

The pseudo-first-order kinetic model, pseudo-second-order kinetic model, Elovich, and Weber–Morris models were used to simulate the adsorption kinetics of Cr(VI) on the adsorbent. The equations associated with the above four models are listed as follows:

(3) $$\mathrm{Pseudo-first-order}:\;\log(Q_{e}\;-\;Q_{t})\;=\;\log Q_{e}\;-\;\frac{k_{1}}{2.303}t$$

(4) $$\mathrm{Pseudo-second-order}:\;\frac{t}{\;Q_{t}}=\frac{1}{K_{2}Q_{e}^{2}}+\frac{t}{Q_{e}}$$

(5) $$\mathrm{Elovich}:\;Q_{t}=\frac{1}{\beta }\ln\left(\alpha \beta \right)+\frac{1}{\beta }\ln t$$

(6) $$\mathrm{Weber–Morris}:\;Q_{t}=K_{\mathrm{di}}t^{1/2}+C_{i}$$

where *Q_e_* (mg/g) and *Q_t_* (mg/g) are the adsorption amounts of Cr(VI) at equilibrium and reaction time *t*, respectively. *K*_1_ (min^−1^) and *K*_2_ (g/(mg·min)) are first-order and second-order rate constants, respectively. *α* is the initial adsorption rate constant, mg/(g·min); *β* is the degree of adsorbent surface coverage and the correlation coefficient of chemical adsorption activation energy, g/min. *K_di_* is the diffusion coefficient, mg/(g·min^1/2^); *C_i_* is a constant related to the boundary layer thickness. If the fitted lines for *Q_t_* and *t*^1/2^ pass through the origin (*C_i_* = 0), it indicates that intraparticle diffusion is the rate-controlling step. If the fitted line for *Q_t_* and *t*^1/2^ does not pass through the origin (*C_i_* ≠ 0), it suggests that factors other than intraparticle diffusion are influencing the adsorption process.

#### 3.4.2. Adsorption Isotherms Model

Langmuir, Freundlich, Sips, and Temkin models were used to simulate the adsorption isotherms of Cr(VI) on the adsorbent. The equations of the four models are as follows:

(7) $$\mathrm{Langmuir}:{\;Q}_{e}\;=\;\frac{Q_{m}K_{L}C_{e}}{1\;+\;K_{L}C_{e}}$$

(8) $$\mathrm{Freundlich}:{\;Q}_{e}=K_{F}C_{e}^{1/n}$$

(9) $$\mathrm{Sips}:{\;Q}_{e}=\frac{Q_{m}{\left(K_{s}C_{e}\right)}^{m}}{1+{\;(K_{s}C_{e})}^{m}}$$

(10) $$\mathrm{Temkin}:\;Q_{e}=B_{1}(\ln\;A_{T})+B_{1}(\ln\;C_{e})$$

where *Q_e_* is the adsorption capacity at equilibrium (mg/g), *C*_0_ and *C_e_* are the initial and equilibrium concentrations of Cr(VI) (mg/L), *Q_m_* is the maximum adsorption capacity of the material for Cr(VI) (mg/g), and *K_L_* is the equilibrium constant of the Langmuir model related to the adsorption interaction strength. *K_F_* and 1/*n* are the adsorption equilibrium constant and adsorption strength constant of the Freundlich equation, respectively; *K_s_* is the adsorption equilibrium constant of the Sips equation, and *m* is used to measure the heterogeneity of adsorption sites on the material surface; *A_T_* and *B*_1_ are constants of the Temkin model, where *A_T_* is the Temkin isotherm constant (L·g^−1^) and *B*_1_ is defined by the expression *B*_1_ = *RT*/*b*, where *b* is the Temkin constant (J·mol^−1^), *T* is the absolute temperature (K), and *R* is the gas constant (8.314 J/(mol·K)).

#### 3.4.3. Adsorption Thermodynamics Model

Thermodynamic parameters were calculated using the following equations:

(11) $$\Delta G^{\theta }\;=\;-\mathrm{RT}\ln K_{c}$$

(12) $$K_{c}=\frac{Q_{e}\;}{C_{e}}$$

(13) $$\Delta G^{\theta }=\Delta H^{\theta }-T\Delta S^{\theta }$$

(14) $$\ln\;K_{c}=-(\frac{\Delta H^{\theta }}{\mathrm{RT}})+\frac{\Delta S^{\theta }}{R}$$

where $\Delta G^{\theta }$ is the change in Gibbs free energy, kJ/mol; *T* is the Kelvin temperature, K; *R* is the universal gas constant, 8.314 J/(mol·K); *K_c_* is the thermodynamic equilibrium constant; *Q_e_* is the adsorption capacity at equilibrium, mg/g; *C_e_* is the adsorption capacity at equilibrium, mg/g; $\Delta H^{\theta }$ is the standard enthalpy change, kJ/mol; and $\Delta S^{\theta }$ is the standard entropy change, J/(mol·K).

### 3.5. Analytical Techniques

Referring to the reported method used for Cr(VI) detection [107], the concentration of Cr(VI) in aqueous solutions was determined by an inductively coupled plasma optical emission spectrometer (Optima 7000DV, Platinum Elmer Instruments, Inc., Waltham, MA, USA). The crystal structure was determined by X’Pert3 Powder-type multifunctional XRD (Panaco, London, UK, copper target, λ = 1.54056 Å). The scanning step, speed, and range were 0.02626°, 0.6565°/s, and 5–90° (2θ), respectively. The functional groups were measured using IS10 FT-IR (Thermo Fisher, Waltham, MA, USA). The specific surface area (S_BET_) and average pore diameter were determined by a BET analyzer (Quantachrome Nova 4200e, Boynton Beach, FL, USA). The thermal stability was evaluated using Thermogravimetric analysis (TGA) (HITACHI STA200/300, Tokyo, Japan). The magnetic property was characterized using VSM (Lake Shore 8604, Westerville, OH, USA). The surface morphology and elemental analysis were determined by SEM-EDS (Zeiss Sigma300, Oberkochen, Germany). The chemical and electronic states of the elements were analyzed using XPS (Escalab 250Xi, Thermo Fisher, Waltham, MA, USA).

## 4. Conclusions

In this study, Fe-TA-CSB has been successfully synthesized by modifying corn straw-based biochar with magnetite and tannic acid (m(TA):m(Fe_3_O_4_) = 12.5%), and then is applied in the removal of Cr(VI) from acidic aqueous solutions. The findings found that Fe-TA-CSB exhibits a higher value of removal efficiency (*R_e_*) of Cr(VI) (94.02%) compared to control materials such as Fe-CSB (86.53%) and CSB (63.22%), and the optimal experimental conditions for Cr(VI) removal are determined to be pH = 3, *m*/*v* = 1.0 g/L, and initial concentrations (*C*_0_) of 10 mg/L. The co-existing cations such as ${\mathrm{Mg}}^{2+}$, ${\mathrm{Ca}}^{2+}$, ${\mathrm{Na}}^{+}$, ${\mathrm{CO}}_{3}^{2-}$, and ${\mathrm{SO}}_{4}^{2-}$, have an inhibiting effect on the removal of Cr(VI) with the decrease in the *R_e_* values of 6.75–10.50%. The adsorption process of Cr(VI) by Fe-TA-CSB is well fitted to the Elovich kinetics model and Sips isotherm model, with a maximum adsorption capacity of 17.89 mg/g. The thermodynamic experiments suggest that the Cr(VI) adsorption is spontaneous and endothermic. Characterization analysis of XRD, FTIR, SEM and XPS indicates that rich pore structure and iron polyphenol complex of Fe-TA-CSB are the main active adsorption sites of Cr(VI), making Cr(VI) adsorbed on the surface of Fe-TA-CSB. Then, Cr(VI) can be effectively removed by the synergistic reduction and complexation through Fe–O, O–H, and C–O and other functional groups.

## Figures and Tables

**Table 1:** Content of organic elements of CSB, Fe-CSB, and Fe-TA-CSB.

Sample	Atomic Ratio of Elements (%)				H/C	O/C
	C	H	O	N		
CSB	55.11	2.59	7.90	1.12	0.047	0.143
Fe-CSB	35.90	2.50	10.88	0.80	0.070	0.300
Fe-TA-CSB	36.86	3.28	8.54	0.74	0.080	0.232

**Table 2:** The parameters of CSB, Fe-CSB, and Fe-TA-CSB.

Sample	SSA (m^2^/g)	PV (cm^3^/g)	D_m_ (nm)	D_max_ (nm)	
				Adsorption	Desorption
CSB	253.604	0.1265	1.996	3.183	3.832
Fe-CSB	231.774	0.1327	2.29	3.173	3.628
Fe-TA-CSB	250.370	0.1421	2.27	3.268	3.922

**Table 3:** Adsorption kinetics model parameters.

Kinetics Model	Parameters		Temperature (°C)
			25
Pseudo First Order	*Q*_e_ (mg/g)		8.78
	*K*_1_		0.0284
	*R* ^2^		0.8408
Pseudo Second Order	*Q*_e_ (mg/g)		8.95
	*K*_2_		0.0047
	*R* ^2^		0.8893
Elovich	*α*		7.911
	*β*		1.101
	*R* ^2^		0.9939
Weber–Morris	0–360 min	*K_d_*_1_ (mg/(g·min^1/2^)	0.1369
		*C*_1_	4.3798
		*R* ^2^	0.9626
	540–1440 min	*K_d_*_2_ (mg/(g·min^1/2^)	0.0674
		*C*_2_	5.9375
		*R* ^2^	0.9634
	2160–3600 min	*K_d_*_3_ (mg/(g·min^1/2^)	0.0392
		*C*_3_	7.1592
		*R* ^2^	0.9653

**Table 4:** Adsorption isotherm fitting data table.

Isotherms Model	Parameters	Temperature (°C)		
		25	35	45
Langmuir	*Q_m_* (mg/g)	17.89	19.18	20.34
	*K_L_* (L/mg)	1.807	1.630	1.589
	*R* ^2^	0.9998	0.9934	0.9908
Freundlich	*K_F_*	8.900	9.504	9.972
	1/*n*	0.2202	0.2150	0.2193
	*R* ^2^	0.8881	0.9108	0.9155
Sips	*Q_m_* (mg/g)	17.89	19.18	20.35
	*K_s_*	1.836	1.812	1.772
	*m*	0.9733	0.8226	0.8093
	*R* ^2^	0.9938	0.9908	0.9903
Temkin	*A_T_* (L/mg)	39.95	72.36	79.92
	*B*_1_ (J/mol)	2.649	2.502	2.631
	*R* ^2^	0.9419	0.9530	0.9531

**Table 5:** Adsorption thermodynamics data table.

Temperature/K	K_c_	ΔG^θ^/kJ/mol	ΔS^θ^/J/(mol·K)	ΔH^θ^/kJ/mol
298	15.71	−6.825	64.62	12.42
308	18.65	−7.492		
318	21.54	−8.116		

**Table 6:** Comparison of Cr(VI) removal by Fe-TA-CSB and other similar composites.

Adsorbents	pH	T (°C)	*C*_0_ (mg/L)	*m*/*v* (g/L)	*Q_m_* (mg/g)	Reusability	Reference
TP-SSA (tea polyphenols modified iron-containing sludge by pyrolysis impregnation method)	2.0	25	0.5–5	1.0	2.52	-	[84]
MHDB (FeCl_3_-modified honeysuckle residue biochar by impregnation pyrolysis method)	2.0	25	10–100	2.0	9.95	-	[85]
m-biochar (nano-magnetite modified reed-derived biochar by situ microwave synthesis method)	3.0	25	20	2.0	9.92	-	[86]
GSMB (FeCl_3_-modified white tea waste biochar by aqueous chemical reduction method)	3.0	25	3–150	2.0	9.11	-	[87]
nZVI modified sludge biochar by the co-pyrolysis method	2.0	25	50	4.0	13.27	-	[88]
BMBC (magnetite rice straw biochar modified by cystamine and glutaraldehyde)	2.0	30	10–500	2.0	66.10	5 times, 61.12%	[89]
MFBAP (HAD-modified magnetic biochar by hydrothermal method)	2.0	25	20–500	1.0	80.58	5 times, 33.68 mg/g	[90]
Fe-TA-CSB	3.0	25	0.5–50	1.0	17.89	4 times, 81.14%	This work

## Data Availability

All data used during the study appear in the article.
